# Is There Proof of Extraskeletal Benefits From Vitamin D Supplementation From Recent Mega Trials of Vitamin D?

**DOI:** 10.1002/jbm4.10459

**Published:** 2021-01-04

**Authors:** Robert Scragg, John D Sluyter

**Affiliations:** ^1^ School of Population Health University of Auckland Auckland New Zealand

**Keywords:** CANCER, CARDIOVASCULAR DISEASE, CLINICAL TRIALS, FALLS PREVENTION, FRACTURE PREVENTION, RESPIRATORY INFECTION, VITAMIN D SUPPLEMENTATION

## Abstract

Scientific interest in possible extraskeletal effects of vitamin D first appeared in the 1930s soon after the structure of vitamin D was characterized, and increased in the 1980s with the development of assays of 25‐hydroxyvitamin D status as a marker of vitamin D status, which in observational epidemiological studies was shown to be inversely associated with many nonskeletal diseases. This resulted in the start of seven large randomized controlled trials (*n* > 2000 participants in each) of vitamin D supplementation giving higher doses than previously used. The intervention periods in these trials collectively started in 2009 and continued to 2020. They have recruited participants, mostly of both sexes and over the age of 50 years, from many countries and have given either daily or monthly doses of vitamin D. Collectively, the trials have a wide range of outcomes with the main focus on the prevention of cancer, cardiovascular disease, and fractures, besides many other outcomes. The findings of four trials have been published, and they have shown that vitamin D supplementation does not prevent hard‐disease endpoints, such as cardiovascular disease, cancer, fractures, or falls, aside from a possible beneficial effect against cancer mortality. In contrast, beneficial effects were seen for intermediate outcomes such as BMD of spine and hips, arterial function, and lung function, especially in people with vitamin D deficiency. The finding of a benefit primarily in people with vitamin D deficiency, if confirmed by the other trials, would support a population approach to preventing vitamin D deficiency using fortification rather than the high‐risk approach of screening for deficiency combined with supplementation. The findings on other outcomes from the three published trials, along with the findings from the four unpublished trials, are expected within the next 2 to 3 years to clarify the role of vitamin D supplementation in preventing nonskeletal disease. © 2020 The Authors. *JBMR Plus* published by Wiley Periodicals LLC. on behalf of American Society for Bone and Mineral Research.

## Introduction

Several large randomized controlled trials (*n* > 2000 participants in each) of vitamin D supplementation giving higher doses than previously used. The intervention periods in these trials collectively started in 2009 and continued to 2020.

To date, four trials have published their findings and have shown that vitamin D supplementation does not prevent hard‐disease endpoints, such as cardiovascular disease (CVD), cancer, fractures, or falls, aside from a possible beneficial effect against cancer mortality.

In contrast, beneficial effects were seen for intermediate outcomes such as BMD of spine and hips, arterial function, and lung function, especially in people with vitamin D deficiency.

Evidence of extraskeletal effects from vitamin D extends back to the beginning of the 20th century when the Danish physician, Niels Finsen, used sunlight to treat lupus vulgaris by concentrating it locally on the skin lesions of this disease.^(^ ^1^ ^)^ The success of this treatment, relative to other treatments at that time, was acknowledged by the awarding of the Nobel Prize to him in 1903.^(^ ^2^ ^)^ Around the same time, Swiss physicians Oskar Bernhard and Auguste Rollier started treating patients suffering from surgical tuberculosis with whole‐body sun exposure, culminating in the development of clinics in the Swiss Alps for the treatment of this disease.^(^ ^2^ ^)^

The observation of antirachitic properties from food exposed to ultraviolet (UV) radiation in 1924 by Steenbock,^(^ ^3^ ^)^ and the isolation and characterization of the structure of vitamin D by 1932,^(^ ^4^, ^5^ ^)^ combined with the knowledge of the partial success of sunlight in treating tuberculosis, led to the use of oral vitamin D to treat lupus vulgaris up until the late 1940s.^(^ ^6^ ^)^ It was only with the success of antibiotics such as streptomycin in the treatment of pulmonary tuberculosis^(^ ^7^ ^)^ that treatment of this disease with vitamin D waned in the next decades and was subsequently forgotten.

Scientific interest in the possible extraskeletal effects of vitamin D re‐emerged in the 1980s. Work by Anthony Norman and colleagues showed that treatment with vitamin D increased insulin secretion in vitamin D‐deficient rats.^(^ ^8^ ^)^ This was extended by ecological epidemiological studies describing inverse associations of mortality from colorectal cancer and CVD with UV radiation through latitude and season.^(^ ^9^, ^10^ ^)^ Both studies suggested a role for vitamin D status in determining the risk of these diseases given the critical role of UV‐B light in the dermal synthesis of vitamin D.^(^ ^11^ ^)^

The development of assays in the 1970s to measure the main metabolite of vitamin D, 25‐hydroxyvitamin D [25(OH)D], which could be used as a marker of vitamin D status,^(^ ^12^ ^)^ enabled the conduct of observational epidemiological studies to identify nonskeletal diseases possibly linked to vitamin D status. Evidence that circulating levels of 25(OH)D were inversely associated with risk of colorectal cancer,^(^ ^13^ ^)^ combined with ecological evidence that risk of breast cancer mortality was inversely associated with solar radiation,^(^ ^14^ ^)^ resulted in a calcium–vitamin D supplementation arm being added to the Women's Health Initiative (WHI) to investigate their combined effect on incidence of both cancers and also hip fractures.^(^ ^15^, ^16^, ^17^ ^)^

While the WHI was being conducted, results continued to be published from observational studies showing that vitamin D status was inversely associated with the risk of coronary heart disease,^(^ ^18^ ^)^ diabetes mellitus,^(^ ^19^ ^)^ respiratory infection,^(^ ^20^ ^)^ and levels of blood pressure,^(^ ^21^ ^)^ and positively associated with lung function.^(^ ^22^ ^)^ In many of these observational studies, the inverse associations between vitamin D status and disease risk were linear across the full population range of circulating 25(OH)D, from <20 nmol/L up to >100 nmol/L. These results suggested that most of the population could benefit from increasing their vitamin D status, and drove the need to conduct clinical trials using high doses of vitamin D supplementation to determine whether the associations were causal.

This was reinforced by the null results from the WHI, which only gave 400 IU/d of vitamin D_3_, with this low dose considered to be a reason for the null effect,^(^ ^23^ ^)^ although the range of vitamin D intake would have been greater because participants were allowed to take their own self‐purchased vitamins. This conclusion, combined with potential benefit from higher vitamin D doses along with evidence about the safety of higher‐dose vitamin D,^(^ ^24^ ^)^ resulted in the setting up of the current collection of vitamin D mega trials. The aim of these studies was to determine if higher doses of vitamin D than used previously (typically 400–800 IU/d), taken for several years, could show a wide range of extraskeletal benefits.

These trials started their interventions between 2009 and 2014. However, during their conduct over the last 10 years, evidence has started to emerge from observational studies of nonlinear associations between vitamin D status and disease or mortality risk, with measures of relative risk being increased at 25(OH)D levels below 50 nmol/L and approximating one above this.^(^ ^25^, ^26^, ^27^, ^28^, ^29^ ^)^ This has implications both for the interpretation of the results from the current mega trials and for public policy about increasing vitamin D status, which are discussed below.

## Recent Mega Trials of Vitamin D Supplementation

For this review, we have defined a mega trial as randomizing more than 2000 adult participants. We have included seven from an earlier review,^(^ ^30^ ^)^ after excluding the VIDAL (Vitamin D and Longevity) study that randomized 1615 participants selected from general practices in England into a pilot study for a definitive trial with a planned sample size of 20,000, which is still to be carried out.^(^ ^31^ ^)^ The only other recent published trial we are aware of with more than 2000 participants is the D2d (Vitamin D and Type 2 Diabetes) study (*n* = 2423 randomized), with the primary outcome of diabetes mellitus prevention, which is discussed elsewhere (see Pittas and colleagues^(^ ^32^ ^)^).

Key features of the seven trials are summarized in Table 1. In order of the year their interventions started, they are the CAPS (Clinical Trial of Vitamin D3 to Reduce Cancer Risk in Postmenopausal Women) study carried out in Nebraska,^(^ ^33^ ^)^ ViDA (Vitamin D Assessment) study in New Zealand,^(^ ^34^ ^)^ VITAL (Vitamin D and Omega‐3) trial in the United States,^(^ ^35^ ^)^ FIND (Finnish Vitamin D) trial in Finland, DO‐HEALTH (Vitamin D3–Omega3–Home Exercise–Healthy Ageing and Longevity) trial in five European countries (Switzerland, Germany, Austria, France, and Portugal), TIPS‐3 (The International Polycap Study 3) in nine countries across the globe (India, Philippines, Colombia, Bangladesh, Canada, Malaysia, Indonesia, Tunisia, and Tanzania),^(^ ^36^ ^)^ and D‐Health (Vitamin D Supplementation for Reduction of Mortality and Cancer) in Australia.^(^ ^37^ ^)^ The achieved sample size in the FIND study (*n* = 2495) is greatly reduced from the planned original sample of 18,000^(^ ^30^ ^)^ because of difficulties in recruitment based on the increasing use of vitamin D supplements in Finland at the beginning of the study.^(^ ^38^ ^)^

**Table 1:** Summary of Recent Randomized, Controlled Mega Trials of Vitamin D Supplementation

Trial (link to trial registry)^a^	Sample size	Age range (y)	Sex	Country	Vitamin D dose (IU)	Intervention dates	Primary outcomes	Status
CAPS (clinicaltrials.gov/show/NCT01052051)	2303	≥55	F	United States	2000/d^b^	2009–2015	Cancer	Published^c^
ViDA (www.anzctr.org.au/Trial/Registration/TrialReview.aspx?id=336777)	5108	50–84	M, F	New Zealand	200,000 start, 100,000/mo	2011–2015	CVD, respiratory infection, fractures, falls	Published^c^
VITAL (clinicaltrials.gov/show/NCT01169259)	25,871	M ≥50 F ≥55	M, F	United States	2000/d^d^	2011–2017	Cancer, CVD	Published^c^
FIND (clinicaltrials.gov/show/NCT01463813)	2495	M ≥60 F ≥65	M, F	Finland	1600/d 3200/d	2012–2018	CVD, cancer	Intervention completed
DO‐HEALTH (clinicaltrials.gov/show/NCT01745263)	2157	≥70	M, F	Europe: mostly Switzerland	2000/d^e^	2012–2018	Fractures, muscle function, blood pressure, cognitive decline, infection	Published^c^
TIPS‐3 (clinicaltrials.gov/show/NCT01646437)	5713	M ≥50 F ≥55	M, F	International: mostly India, Philippines	60,000/mo ^f^	2012–2020	Fractures	Intervention ongoing
D‐Health (www.anzctr.org.au/Trial/Registration/TrialReview.aspx?id=364534)	21,315	60–84	M, F	Australia	60,000/mo	2014–2020	All‐cause mortality	Intervention completed

CVD = cardiovascular disease; F = Female; M = Male.

^a^ Accessed May 5, 2020.

^b^ With calcium carbonate 1500 mg/d.

^c^ See Table 2.

^d^ 2 × 2 Factorial design with marine omega‐3 fatty acids 1 g/d.

^e^ 2 × 2 × 2 Factorial design with marine omega‐3 fatty acids 1 g/d; home exercise.

^f^ 2 × 2 × 2 Factorial design with antihypertensives and statin; aspirin.

All studies included participants of both sexes except for the CAPS study that recruited women only. The age range was generally 50 years and above; individuals in this age range have the highest risk of the primary study outcomes, which were mainly cancer, CVD, and fractures.

The vitamin D‐dosing frequency was daily in four studies and monthly in three. All studies specified giving vitamin D as cholecalciferol (vitamin D_3_) except for TIPS‐3, which is assumed to have done the same.^(^ ^36^ ^)^ The actual vitamin D dose was 2000 IU/d (or monthly equivalent), except for the FIND study, which gave daily doses of 1600 IU and 3200 IU in a three‐arm study, and the ViDA study, which gave a double dose (200,000 IU) at randomization followed a month later by 100,000 IU monthly. Four studies were the standard parallel design with vitamin D and placebo arms, except for the VITAL study, which was a 2 × 2 factorial design with marine‐omega‐3 fatty acids, DO‐HEALTH, which was a 2 × 2 × 2 factorial design with marine‐omega‐3 fatty acids and home exercise, and TIPS‐3, which was a 2 × 2 × 2 factorial design with antihypertensives combined with statin and aspirin. Only the CAPS study combined calcium with the vitamin D intervention. All studies have completed recruitment, and the intervention period has been completed in all except for TIPS‐3, which is expected to finish in 2020. To date, only four studies have published results: CAPS, ViDA, VITAL, and DO‐HEALTH.

## Published Results

Table 2 summarizes some key baseline characteristics for the CAPS,^(^ ^33^ ^)^ ViDA,^(39^ ^)^ VITAL,^(40^ ^)^ and DO‐Health^(^ ^41^ ^)^ studies, along with results for both primary and secondary outcomes and from ancillary studies to the ViDA and VITAL trials.

**Table 2:** Published Results From Recent Randomized Controlled Mega Trials of Vitamin D Supplementation

Characteristic	Study			
	CAPS	ViDA	VITAL	DO‐HEALTH
Baseline demographic and clinical				
Age, y: mean (SD)	65.2 (7.0)	65.9 (8.3)	67.1 (7.0)	74.9 (4.4)
Female: *n* (%)	2303 (100)	2139 (41.9)	13,085 (50.6)	1331 (61.7)
Race/ethnicity: *n* (%)	Non‐Hispanic White: 2291 (99.5) Other: 12 (0.5)	Maori: 272 (5.3) Pacific Islander: 334 (6.5) South Asian: 249 (4.9) European/other: 4253 (83.3)	Non‐Hispanic White: 18,046 (71.3) Black: 5106 (20.2) Hispanic: 1013 (4.0) Asian/Pacific Islander: 388 (1.5) Native American: 228 (0.9) Other/unknown: 523 (2.1)	European: 2157 (100)
Current smoking: *n* (%)	141 (6.1)	320 (6.3)	1836 (7.2)	126 (5.8)
Hypertension (treated): *n* (%)	—	1885 (36.9)	12,791 (49.8)	1069 (49.6)
Diabetes mellitus: *n* (%)	—	504 (9.9)	3549 (13.7)	—
BMI (kg/h^2^): Mean (SD)	30.0 (6.6)	28.4 (5.1)	28.1 (5.7)	26.3 (4.3)
25(OH)D (nmol/L):				
Baseline mean (SD)	82 (26)	63 (24)	77 (25)	56 (21)
<25 nmol/L – *n* (%)	32 (1)^a^	161 (3)	486 (2)^a^	153 (7)^a^
25–49 nmol/L – *n* (%)	216 (9)^a^	1373 (27)	3138 (12)^a^	688 (32)^a^
50–74 nmol/L – *n* (%)	658 (29)^a^	2014 (39)	8486 (33)^a^	925 (43)^a^
≥75 nmol/L – *n* (%)	1397 (61)^a^	1558 (31)	13761 (53)^a^	391 (18)^a^
Mean increase in vitamin D group	30 – during follow‐up	69 at 3 y	30 at 1 y	33 at 3 y
Median supplementation period, y	4	3.3	5.3	3.0
Outcomes (reference = placebo)				
Cancer: HR (95% CI)				
Incidence				
All	0.70 (0.47–1.02)	1.01 (0.81–1.25)	0.96 (0.88–1.06)	—
Baseline 25(OH)D <50 nmol/L		1.01 (0.65–1.58)	0.97 (0.28–1.39)	
Mortality	—	0.99 (0.60–1.64)	0.83 (0.67–1.02)	—
Cardiovascular and renal				
Cardiovascular disease: HR (95% CI)				
All	—	1.02 (0.87–1.20)	0.97 (0.85–1.12)	—
Baseline 25(OH)D <50 nmol/L		1.00 (0.74–1.35)	1.09 (0.68–1.76)	
Heart failure: HR (95% CI)	—	1.19 (0.84–1.68)	0.93 (0.78–1.11)	—
Blood pressure: mm Hg, mean difference (99% CI) in change (over 3 y)				
Systolic: All	—	—	—	−0.8 (−2.1, 0.5)
Systolic: Baseline 25(OH)D <50 nmol/L				−1.4 (−3.5, 0.8) *n* = 872
Diastolic: All	—	—	—	0 (−0.7, 0.8)
Arterial function: Augmentation index, %, mean difference in change (at 1 y)				
All	—	0.0% (*n* = 517, *p* = 0.98)	—	
Baseline 25(OH)D <50 nmol/L		−5.7% (*n* = 150, *p* = 0.03)		
GFR: In type 2 diabetes mellitus, mL/min/1.73 m^2^, mean difference in change (at 5 y)				
All	—	—	0.9 (−0.7, 2.5) *n* = 934, *p* = 0.25	—
Baseline 25(OH)D <50 nmol/L			2.4 (−2.1, 6.9) *n* = 195, *p* = 0.29	
Musculoskeletal and mental function				
Falls: HR (95% CI)				
All	Secondary outcome	0.99 (0.92–1.07)	0.97 (0.90–1.05)^b^	
Baseline 25(OH)D <50 nmol/L	(unpublished)	1.07 (0.91–1.25)	1.03 (0.59–1.79)^b^ ^,^ ^c^	
Physical function: SPPB score, mean difference (99% CI) in change (over 3 y)	—	—	—	−0.1 (−0.3, 0.1)
Fractures: HR (95% CI)				
All	Secondary outcome	1.19 (0.94–1.50)	Ancillary study (unpublished)	1.03 (0.75, 1.43)^d^
Baseline 25(OH)D <50 nmol/L	(unpublished)	0.94 (0.58–1.52)		
Spine BMD: % mean difference in change (2 y)				
All	—	0.6% (*n* = 418, *p* = 0.093)	0.16% (*n* = 634, *p* = 0.55)	
Low baseline 25(OH)D		2.6% (*n* = 36, *p* = 0.035)	0.75% (*n* = 320, *p* = 0.043)^e^	
NSAID prescriptions: RR (95% CI)				
All	—	0.97 (0.92–1.02)	—	—
Baseline 25(OH)D <50 nmol/L		0.87 (0.78–0.96)		
Statin persistence: HR (95% CI)				
All	—	1.15 (1.02–1.30)	—	—
Baseline 25(OH)D <50 nmol/L		1.12 (0.89–1.41)		
Depression: HR (95% CI)				
All	—	—	0.97 (0.87–1.09)	
Baseline 25(OH)D <50 nmol/L			1.25 (0.84–1.89)	
Cognitive function: MoCA score, mean difference (99% CI) in change (over 3 y)	—	—	—	−0.1 (−0.4, 0.1)
Respiratory infection and function				
Acute respiratory infection: HR (95% CI)				
All	Secondary outcome	1.01 (0.94–1.07)	Ancillary study (unpublished)	Secondary outcome
Baseline 25(OH)D <50 nmol/L	(unpublished)	1.08 (0.95–1.23)		(unpublished)
FEV_1_: mL, in ever smokers, mean difference in change (at 1 y)				
All		57 mL (*n* = 217, *p* = 0.03)	Ancillary study (unpublished)	—
Baseline 25(OH)D <50 nmol/L	—	122 mL (*n* = 54, *p* = 0.04)		

25(OH)D = 25‐hydroxyvitamin D; CAPS = Clinical Trial of Vitamin D3 to Reduce Cancer Risk in Postmenopausal Women; DO‐HEALTH = Vitamin D3–Omega3–Home Exercise–Healthy Ageing and Longevity; FEV_1_ = forced expiratory volume in 1 s; GFR = glomerular filtration rate; HR = hazard ratio; MoCA = Montreal Cognitive Assessment; NSAID = nonsteroidal anti‐inflammatory drug; RR = relative risk; SPPB = Short Physical Performance Battery; ViDA = Vitamin D Assessment; VITAL. = Vitamin D and Omega‐3.

^a^ Estimated.

^b^ Odds ratio.

^c^ 25(OH)D ≤30 nmol/L.

^d^ Incidence rate ratio, 99% CI.

^e^ Free 25(OH)D <median 14.2 pmol.

### Baseline characteristics

The ethnic/race composition of the four studies varied from being almost exclusively of White/European ancestry in theDO‐HEALTH and CAPS studies, to just over 80% European with the remainder Polynesian and South Asian (about 17%) in the ViDA study, to 71% non‐Hispanic White, 20% Black, and 4% Hispanic in the VITAL study. Only 6% to 7% of study participants were current smokers, whereas treated hypertension was reported by over a third of participants in the ViDA and half in VITAL and DO‐HEALTH trials. Diabetes mellitus also was common, reported by 14% in the VITAL and 10% in the ViDA studies. Mean BMI ranged from 26 to 30, indicating a high prevalence of obesity (BMI ≥30 kg/m^2^) in all four studies. Despite a relatively high mean BMI, the mean baseline 25(OH)D was not low, varying from 56 nmol/L in the DO‐HEALTH study up to 82 nmol/L in the CAPS. The number of participants with severe vitamin D deficiency [25(OH)D <25 nmol/L] in each study was only 32 (1%) in the CAPS, 161 (3%) in the ViDA, 486 (2%) in the VITAL, and 153 (7%) in the DO‐HEALTH studies, whereas insufficiency (25(OH)D <50 nmol/L), respectively, was 248 (11%), 1534 (30%), 3625 (14%), and 841 (39%). These numbers were estimated from published data for the CAPS,^(33^ ^)^ VITAL,^(40^ ^)^ and DO‐HEALTH studies,^(41^ ^)^ and the actual number for observed 25(OH)D in the ViDA trial.^(^ ^39^ ^)^ The mean increase in 25(OH)D during follow‐up in the vitamin D group was 69 nmol/L in the ViDA study—more than twice as high as in the other three studies. This reflects the higher vitamin D dose given in the ViDA study along with the lower baseline 25(OH)D (except for the DO‐HEALTH trial) because the increase in this metabolite is inversely related to baseline vitamin D status as shown in recent publications from both the ViDA and VITAL teams.^(^ ^42^, ^43^ ^)^

### Cancer

Incidence of all types of cancer (excluding nonmelanoma skin cancer) is the only outcome reported by all three trials. The CAPS trial was the first to report.^(^ ^33^ ^)^ This trial found a 1.7% absolute decrease in cancer incidence in the vitamin D‐treated group compared with placebo that just failed to achieve statistical significance (*p* = 0.06) and a 30% relative decline in the hazard ratio (HR; Table 2). In a post hoc analysis, the HR was significantly decreased in the vitamin D group for cancer cases detected 12 months or more after randomization (0.65; 95% CI, 0.42‐0.99; *p* = 0.046). This result was of interest because it replicated findings from an earlier trial by the same group.^(^ ^44^ ^)^

In contrast, none of the results in the CAPS study were confirmed by those from the ViDA^(^ ^45^ ^)^ or VITAL trials,^(40^ ^)^ which reported HRs very close to 1.00 for nearly all cancer outcomes, including in participants with baseline 25(OH)D <50 nmol/L in both studies and for site‐specific cancers (breast, prostate, and colorectal) in the VITAL study. The difference in the results between CAPS and the other two studies could be caused by the cointervention with calcium in the CAPS in contrast with the ViDA and VITAL studies, which gave vitamin D alone. However, the WHI did not see a beneficial effect against cancer incidence when combining vitamin D with calcium, although its dose of vitamin D (400 IU/d) may have been too low.^(^ ^46^ ^)^ The use of monthly bolus vitamin D dosing is unlikely to be an explanation for the lack of effect seen in the ViDA study because its results for cancer incidence are no different to those from the VITAL trial.

The main outcome of interest was a 17% decrease in the HR for cancer mortality among the vitamin D‐treated group in the VITAL trial. This outcome has been found to be significantly reduced by 13% (relative risk, 0.87; 95% confidence intervals, 0.79–0.96) in a recent meta‐analysis combining results from previous mega trials.^(^ ^47^ ^)^

### Cardiovascular and renal outcomes

Although CVD is a primary outcome for the ViDA, VITAL, and FIND trials, it has only been reported by the two former studies, possibly because of the greatly reduced sample size of FIND, down from 18,000 originally planned to 2495 achieved (as mentioned above).

Vitamin D supplementation did not reduce the cumulative incidence of all CVD (International Classification of Diseases codes I10‐I82, 10th revision) in the ViDA study^(39^ ^)^ or of major cardiovascular events (combined myocardial infarction, stroke, and death from CVD) in the VITAL trial,^(^ ^40^ ^)^ with HRs being very close to 1.00 in all participants and also in those with baseline 25(OH)D <50 nmol/L. There was also no beneficial effect from vitamin D seen in relation to specific CVDs, including heart failure in the main ViDA study,^(39^ ^)^ in an ancillary study to the VITAL trial,^(^ ^48^ ^)^ and in relation to blood pressure in the DO‐HEALTH study.^(^ ^41^ ^)^ The latter result is consistent with an individual patient data meta‐analysis.^(^ ^49^ ^)^

The effect of vitamin D on arterial function, a risk factor for CVD,^(^ ^50^ ^)^ has been studied in the ViDA study. In a random sample of participants, there was no significant difference between vitamin D and placebo in the mean change in several measures of arterial function and stiffness measured at baseline and 1 year after randomization. However, in participants with baseline 25(OH)D <50 nmol/L, several measures (including augmentation index, pulse wave velocity, and aortic systolic blood pressure, among others) were significantly decreased compared with placebo.^(^ ^51^ ^)^ There was an interaction with the reduction in arterial function measures being significantly greater for augmentation index and pulse wave velocity among vitamin D‐deficient participants than those who were not (*p*_interaction_ < 0.05). These findings are consistent with those of a recent meta‐analysis of randomized controlled trials, which found that vitamin D supplementation lowered carotid‐femoral pulse wave velocity, particularly when vitamin D was given in a dose of ≥2000 IU/d for at least 4 months.^(^ ^52^ ^)^

The effect of vitamin D on glomerular filtration rate in participants with type 2 diabetes mellitus has been reported in an ancillary study to the VITAL study. The mean difference in change over 5 years in the vitamin D compared with placebo was 0.9 mL/min/1.73 m^2^ (*p* = 0.25). A larger mean difference in change (2.4 mL/min/1.73m^2^) was seen in participants with baseline 25(OH)D <50 nmol/L, although the result was not significant (*p* > 0.05).^(^ ^53^ ^)^

The overall findings for CVD indicate that vitamin D supplementation does not prevent this disease group, a conclusion confirmed by a recent meta‐analysis of randomized controlled trials.^(^ ^54^ ^)^

### Musculoskeletal and mental function

The ViDA study is the first of the recent mega trials so far to report on the effect of vitamin D on falls prevention.^(^ ^55^ ^)^ The study found that vitamin D did not reduce the cumulative risk of each participant having one (or more) fall during the follow‐up period, either in the total sample or in those with 25(OH)D <50 nmol/L (Table 2). Recent results from the VITAL study are confirmatory, with no effect from vitamin D on the odds of having two or more falls during follow‐up seen in the total sample or in participants with baseline 25(OH)D ≤30 nmol/L (Table 2).^(^ ^56^ ^)^ The evidence from these two studies strongly suggests that vitamin D supplementation by itself does not prevent falls among people dwelling in the community, even in people who are vitamin D deficient. Other current mega trials—CAPS, DO‐HEALTH, TIPS‐3, and D‐Health—have falls as a secondary outcome (Supplemental Table S1), whereas the DO‐HEALTH study has recently reported that vitamin D does not improve the Short Physical Performance Battery score.^(^ ^41^ ^)^ Further publications from these studies should provide final clarity about whether vitamin D helps to prevent falls by community‐living people and to reduce the rate of falls by people living in care homes.^(^ ^57^ ^)^

The ViDA study is the only recent mega trial to report on fractures as an outcome. Again, there was no effect from vitamin D on this outcome, whether in the total sample or in those with 25(OH)D <50 nmol/L (Table 2).^(^ ^55^ ^)^ These results are consistent with findings from a recent meta‐analysis that also included the ViDA study findings.^(^ ^58^ ^)^ Some of the other current mega trials, CAPS, VITAL, and D‐Health, have fracture as an outcome (Supplemental Table S1) and are expected to provide further results in the near future.

BMD was examined in an ancillary study of the ViDA trial. After 2 years of vitamin D supplementation, no effect on total bone density was seen among all 418 participants in the ancillary study.^(^ ^59^ ^)^ However, in subgroup analyses by baseline 25(OH)D, there were beneficial effects on spine and femoral sites (but not total BMD) only among participants with baseline 25(OH)D ≤30 nmol/L and not in those above this level. A reanalysis of an earlier trial that gave participants daily vitamin D found a similar effect after 1 year of follow‐up.^(^ ^60^ ^)^ Moreover, a VITAL ancillary study with a 2‐year follow‐up, while not confirming a beneficial effect from vitamin D in participants with baseline total 25(OH)D ≤30 nmol/L, did find a benefit in relation to spine and total hip BMD in participants with free 25(OH)D <median 14.2 pmol/L.^(^ ^61^ ^)^ It is unclear why a benefit from vitamin D should be seen for BMD but not fracture prevention in people with vitamin D deficiency. One possible explanation is the effect on BMD was small (only 2% over 2 years) and restricted to the spine and hips. If the effect is cumulative over many years, it is possible that previous studies, including the ViDA study, did not have enough people with very low vitamin D levels who were followed for a long enough period to show a benefit against fractures only in bones of the spine and hip. All fracture‐prevention studies, which gave vitamin D by itself (*n* = 17), only supplemented for a median period of 1 year and a maximum of 5 years.^(^ ^58^ ^)^ This period may not be long enough to see a beneficial effect from vitamin D.

A possible link between vitamin D deficiency and pain has been known for many years.^(^ ^62^ ^)^ This possibility was strengthened by a recent meta‐analysis of clinical trials, which found that pain scores were improved by vitamin D supplementation.^(^ ^63^ ^)^ The ViDA study examined whether vitamin D decreased the prescribing of analgesic drugs for its participants using prescription data provided by the Ministry of Health. Although vitamin D did not decrease the number of participants in the total sample prescribed either opioids or nonsteroidal anti‐inflammatory drugs (NSAIDs), it did decrease the proportion prescribed NSAIDs in participants with baseline 25(OH)D <50 nmol/L.^(^ ^64^ ^)^ Of the other mega trials, the VITAL, DO‐HEALTH, and D‐Health studies all plan to look at pain as an outcome (Supplemental Table S1).

Myalgia is a common outcome in patients prescribed statins,^(^ ^65^ ^)^ and patients with myalgia have lower circulating 25(OH)D levels compared with those without.^(^ ^66^ ^)^ For these reasons, the effect of vitamin D on statin adherence (defined as taking statins >80% of days) and persistence (defined as having gaps of < 30 days between statin prescriptions) over 24 months was examined in the ViDA study. Although vitamin D did not affect adherence in taking statins, it did improve persistence in taking them (Table 2). This finding has clinical significance because the number needed to treat over 24 months to prevent one person stopping their statin medication was 23. Although none of the other mega trials plan to look at this outcome, it clearly requires replication because of its potential importance for patients on statins.

The presence of vitamin D receptors in regions of the brain linked to the pathophysiology of depression,^(^ ^67^ ^)^ along with evidence from observational studies showing increased risk of depression in people with vitamin D deficiency,^(^ ^68^ ^)^ has stimulated the conduct of randomized controlled trials (RCTs) to determine whether vitamin D supplementation prevents depression and improves mood. Most RCTs have reported no beneficial effect from vitamin D, possibly because of their small sample sizes.^(^ ^69^ ^)^ However, a recent publication from the VITAL study, which included 18,353 participants (after exclusions) followed for a median period of 5.3 years with annual assessments of mood by questionnaire, has ensured sufficient power to answer this question. No effect was seen in the total study sample or in those participants with baseline 25(OH)D <50 nmol/L (Table 2).^(^ ^69^ ^)^ This finding, consistent with a mendelian randomization study,^(^ ^70^ ^)^ indicates that vitamin D does not prevent depression. The DO‐HEALTH study also observed that vitamin D did not improve cognitive function as measured by the Montreal Cognitive Assessment score.

### Respiratory infection and function

Given the aforementioned link between sun exposure, vitamin D, and tuberculosis, laboratory evidence that vitamin D increased synthesis of the antimicrobial peptide cathelicidin^(^ ^71^ ^)^ and observational evidence of an inverse association between vitamin D status and risk of respiratory infection symptoms,^(^ ^20^ ^)^ one of the main aims of the ViDA study was to examine the effect of vitamin D supplementation on respiratory infection. The bolus dose of vitamin D used in the ViDA study was found to have no effect on the cumulative risk of acute respiratory infections, both in the total study sample and in participants with baseline 25(OH)D <50 nmol/L.^(^ ^72^ ^)^ This result was consistent with a recent individual‐patient‐data meta‐analysis of RCTs, which found that daily or weekly vitamin D supplementation, but not bolus doses, prevented acute respiratory infection, especially in participants with severe vitamin D deficiency [25(OH)D <25 nmol/L].^(^ ^73^ ^)^ However, a recent large trial of 8851 Mongolian children found that a weekly vitamin D dose of 14,000 IU taken over 3 years did not prevent acute respiratory or tuberculosis infections, even in those with baseline 25(OH)D <25 nmol/L.^(^ ^74^ ^)^ Other trials that have included respiratory infections as an outcome include the CAPS, VITAL, and DO‐Health studies, which gave a daily vitamin D dose, and D‐Health, which gave a monthly bolus dose (Supplemental Table S1). Their results when published are expected to provide further important information about the effect of vitamin D in preventing acute respiratory infections. The DO‐HEALTH found no protective effect from vitamin D against all types of infections (incidence rate ratio, 0.95; 99% CI, 0.84‐1.08).^(^ ^41^ ^)^

Given the observational evidence that vitamin D status is positively associated with spirometric measures of lung function,^(^ ^22^ ^)^ the effect of vitamin D on these measures was examined in a subgroup of the ViDA study. In all participants in this substudy, vitamin D had no effect on either forced vital capacity (FVC) or forced expiratory volume in 1 second (FEV_1_) after 1 year of follow‐up.^(^ ^75^ ^)^ However, a beneficial increase in FEV_1_ was seen in smokers, especially in those with baseline 25(OH)D <50 nmol/L (Table 2). The FEV_1_ increase of 122 mL in the latter group is large enough to be of clinical significance. The only mega trial planning to look at this outcome is the VITAL study,^(^ ^76^ ^)^ and its results are awaited with interest.

## Discussion

The main finding to date from the four recent mega trials that have published some of their results—the CAPS, ViDA, VITAL, and DO‐HEALTH—is that vitamin D supplementation does not prevent hard‐disease endpoints, such as CVD, cancer, or fractures, aside from a possible beneficial effect against cancer mortality.^(^ ^47^ ^)^ No effect also was seen in the ViDA and VITAL trials against falls, even in people with vitamin D deficiency (Table 2).

In contrast, beneficial effects have been seen for intermediate outcomes such as BMD of spine and hips, arterial function, and lung function, especially in people with vitamin D deficiency. The ViDA study has additionally found that vitamin D decreases the prescription of NSAIDs in people with vitamin D deficiency and also increases the continued use of statins (Table 2).

Clarity regarding the recent findings from CAPS, ViDA, VITAL, and DO‐HEALTH are expected within the next 2 to 3 years with further publications on other outcomes from these studies, as well as from the other mega trials, especially the largest of these, the soon to‐be‐completed D‐Health study (Table 1, Supplemental Table S1).

Nevertheless, there are some important issues that need to be considered when evaluating the recent results from these mega trials. First, given the evidence from observational studies of nonlinear associations between vitamin D status and disease risk (cited above), and the implication that vitamin D supplementation will only be beneficial (if at all) in people with vitamin D deficiency,^(^ ^77^ ^)^ the four completed mega trials had very small numbers of people with severe vitamin D deficiency [baseline 25(OH)D <25 nmol/L; Table 2]—too small to detect effects, especially if they are weak. Second, the trials allowed participants in both intervention and placebo arms to continue to take vitamin D supplements (up to 800 IU daily) during follow‐up. The effect of this self‐purchased supplementation on 25(OH)D concentration would have been captured in those who provided a blood sample at baseline. However, given the inverse association between vitamin D status and 25(OH)D response to a standard dose of vitamin D supplements, the commencement of taking self‐purchased vitamin D supplements during follow‐up would have resulted in the greater relative increase in vitamin D status for those in the placebo arm who were vitamin D deficient than those with higher 25(OH)D levels. The net effect of this would have been to attenuate the difference in 25(OH)D concentrations between the vitamin D and placebo arms primarily in those who were vitamin D deficient at baseline, further diminishing the number of participants with vitamin D deficiency in the placebo arm.^(^ ^78^ ^)^ Third, the follow‐up period for the completed trials may have been too short (maximum of 6 years) to detect beneficial effects from vitamin D, particularly for cancer, CVD, and fracture prevention. This possibility is supported by data from the WHI, which did not observe a reduction in hip fracture from combined vitamin D and calcium during the active intervention phase of the study,^(^ ^17^ ^)^ but did find at 11‐year follow‐up decreased vertebral fractures among women randomized to vitamin D and calcium compared with placebo^(^ ^79^ ^)^ and decreased hip fractures in the intervention arm among those who did not report taking vitamin D or calcium supplements at baseline.^(^ ^80^ ^)^ The relatively short follow‐up period could also explain the inconsistent findings of no beneficial effect against the latter hard‐disease endpoints versus the beneficial effects seen for intermediate outcomes linked to these diseases (arterial function, bone density).

The accumulating evidence that vitamin D deficiency is associated with increased risk of disease and with beneficial effects from vitamin D supplementation has implications for preventing and managing diseases linked to vitamin D deficiency. It supports a population approach to prevention, which aims to increase the vitamin D status of the total population through population‐wide strategies such as vitamin D fortification of food and increased but safe sun exposure, rather than the high‐risk approach of screening for vitamin D deficiency and then offering treatment with vitamin D supplementation that is more expensive. Although once detected, severe vitamin D deficiency [25(OH)D <25 nmol/L] should be treated with vitamin D supplements given its accepted role in preventing rickets and osteomalacia,^(^ ^81^ ^)^ regardless of any potential extraskeletal benefits.

In conclusion, recent vitamin D mega trials have not found beneficial effects against hard‐disease endpoints, although benefits have been seen for some intermediate outcomes. Further results from these trials are expected to clarify within the next 2 to 3 years the extraskeletal benefits from vitamin D.

## Disclosures

The authors declared no conflicts of interest.

### Peer Review

The peer review history for this article is available at https://publons.com/publon/10.1002/jbm4.10459.

## Supporting information


**Appendix S1**: Supplementary Information
