# Supplementary material for: Is There Proof of Extraskeletal Benefits From Vitamin D Supplementation From Recent Mega Trials of Vitamin D?
Source: JBMR Plus. 2021 Jan 4;5(1):e10459. doi: 10.1002/jbm4.10459 (PMC7839821; doi:10.1002/jbm4.10459)
Supplement: Supplementary file 1 — Appendix S1: Supplementary Information [file JBM4-5-e10459-s001.docx]

**Online Supplement**

**IS THERE PROOF OF EXTRA-SKELETAL BENEFITS FROM VITAMIN D SUPPLEMENTATION FROM RECENT MEGA-TRIALS OF VITAMIN D?**

Robert Scragg, John D Sluyter

School of Population Health, University of Auckland, Private Bag, Auckland, New Zealand

Supplemental Table 1: Unpublished secondary outcomes in recent randomized controlled mega-trials of vitamin D supplementation *

| **Trial** | **Secondary Outcome** |
| --- | --- |
| CAPS  ([clinicaltrials.gov/show/NCT01052051](http://clinicaltrials.gov/show/NCT01052051) | Breast cancer; colorectal cancer; diabetes mellitus; asthma exacerbations |
| VITAL ^(1)^  ([www.vitalstudy.org/Studies.html](http://www.vitalstudy.org/Studies.html))  [accessed 30 May 2020] | Diabetes; high blood pressure; memory loss or cognitive decline; autoimmune conditions such as thyroid disease, rheumatoid arthritis, and lupus; physical disability; anemia; eye problems such as macular degeneration and dry eye syndrome; COPD & asthma exacerbations; ^(2)^ impaired kidney function in people with high blood pressure; chronic knee pain symptoms; atrial fibrillation |
| FIND  ([clinicaltrials.gov/show/NCT01463813](http://clinicaltrials.gov/show/NCT01463813)) | None planned |
| DO-HEALTH  ([clinicaltrials.gov/show/NCT01745263](http://clinicaltrials.gov/show/NCT01745263)) | Many including:  Bone mineral density; falls; respiratory infections; knee pain (in osteoarthritis); NSAID use; dental health; fasting glucose and insulin; body composition; kidney function; quality of life; mortality; cardiovascular disease; cancer |
| TIPS-3  ([clinicaltrials.gov/show/NCT01646437](http://clinicaltrials.gov/show/NCT01646437)) | CVD; cancer; falls |
| D-Health ^(3)^  (www.anzctr.org.au/Trial/Registration/  TrialReview.aspx?id=364534) | Total cancer; colorectal cancer; CVD; depression; upper respiratory illnesses; COPD & asthma exacerbations;  hyper- or hypothyroidism; diabetes; high blood  pressure; falls; fractures; arthritis; NSAID use; antibiotic use; cognitive decline; health status; muscle aches and pains |

* In addition to those shown in Table 2 for CAPS and VITAL.

COPD = chronic obstructive pulmonary disease CVD = cardiovascular disease

NSAID = non-steroidal anti-inflammatory drug

1. Pradhan AD, Manson JE. Update on the Vitamin D and OmegA-3 trial (VITAL). ***J Steroid Biochem Mol Biol***. Jan 2016;155(Pt B):252-6.

2. Gold DR, Litonjua AA, Carey VJ, Manson JE, Buring JE, Lee IM, et al. Lung VITAL: Rationale, design, and baseline characteristics of an ancillary study evaluating the effects of vitamin D and/or marine omega-3 fatty acid supplements on acute exacerbations of chronic respiratory disease, asthma control, pneumonia and lung function in adults. ***Contemp Clin Trials***. Mar 2016;47:185-95.

3. Waterhouse M, English DR, Armstrong BK, Baxter C, Duarte Romero B, Ebeling PR, et al. A randomized placebo-controlled trial of vitamin D supplementation for reduction of mortality and cancer: Statistical analysis plan for the D-Health Trial. ***Contemp Clin Trials Commun***. Jun 2019;14:100333.
